# Adsorption of Malachite Green and Alizarin Red S Dyes Using Fe-BTC Metal Organic Framework as Adsorbent

**DOI:** 10.3390/ijms22020788

**Published:** 2021-01-14

**Authors:** Giulia Rossella Delpiano, Davide Tocco, Luca Medda, Edmond Magner, Andrea Salis

**Affiliations:** 1 Dipartimento di Scienze Chimiche e Geologiche, Università di Cagliari, Cittadella Universitaria, S.S. 554 bivio Sestu, 09042 Monserrato (CA), Italy; delpiano@unica.it (G.R.D.); davide.tocco@unica.it (D.T.); 2 Laboratorio NEST Scuola Normale Superiore di Pisa, 56127 Pisa, Italy; medda.luc@gmail.com; 3 Department of Chemical Sciences, Bernal Institute, University of Limerick, Limerick V94 T9PX, Ireland; 4 Consorzio Interuniversitario per lo Sviluppo dei Sistemi a Grande Interfase (CSGI), Unità Operativa University of Cagliari, 09042 Monserrato (CA), Italy

**Keywords:** metal organic frameworks, wastewater remediation, adsorption, malachite green, alizarin red S

## Abstract

Synthetic organic dyes are widely used in various industrial sectors but are also among the most harmful water pollutants. In the last decade, significant efforts have been made to develop improved materials for the removal of dyes from water, in particular, on nanostructured adsorbent materials. Metal organic frameworks (MOFs) are an attractive class of hybrid nanostructured materials with an extremely wide range of applications including adsorption. In the present work, an iron-based Fe-BTC MOF, prepared according to a rapid, aqueous-based procedure, was used as an adsorbent for the removal of alizarin red S (ARS) and malachite green (MG) dyes from water. The synthesized material was characterized in detail, while the adsorption of the dyes was monitored by UV-Vis spectroscopy. An optimal adsorption pH of 4, likely due to the establishment of favorable interactions between dyes and Fe-BTC, was found. At this pH and at a temperature of 298 K, adsorption equilibrium was reached in less than 30 min following a pseudo-second order kinetics, with k″ of 4.29 × 10^−3^ and 3.98 × 10^−2^ g∙mg^−1^ min^−1^ for ARS and MG, respectively. The adsorption isotherm followed the Langmuir model with maximal adsorption capacities of 80 mg∙g^−1^ (ARS) and 177 mg∙g^−1^ (MG), and K_L_ of 9.30·10^3^ L∙mg^−1^ (ARS) and 51.56·10^3^ L∙mg^−1^ (MG).

## 1. Introduction

Synthetic organic dyes are among the most harmful polluting agents. It is estimated that 80,000 tons of dyes are produced and consumed each year [1]. They are cheap, offer a wide range of colors, and are used for numerous applications in the paper, tanning, pharmaceutical, photographic, and cosmetic industries [2]. However, synthetic dyes are mainly earmarked for the textile industry, as they possess reactive groups which have a strong binding ability for fiber [3]. The colors of dye molecules are due to chromogenic groups which absorb visible light. Indeed, dye molecules generally have a complex aromatic structure which is often characterized by a high chemical stability. Unfortunately, dyes are highly toxic and can have carcinogenic and mutagenic effects on living organisms, even at low concentrations [4]. In addition, due to their ability to absorb light, the release of dyes into surface waters also causes unwanted effects in the aquatic ecosystem. These effects arise from a reduced level of penetration of the sun’s rays in water, which alters photosynthetic cycles and reduces the oxygen supply in the water body [5]. Due to their high chemical stability, the removal of dyes from water is a challenging issue [6]. Numerous methods have been proposed to remove dyes from wastewaters, such as electrochemical degradation [7,8], membrane-based separation [9], ultrafiltration [10], extraction [11], and biological treatment [12]. While these methods have a number of advantages, they cannot be applied on a large scale due to high costs, secondary pollution, production of waste, etc [3].

Adsorption is a simple method of dye removal that has significant advantages. Indeed, it can be applied to almost any type of dye or mixtures of dyes, it does not require any special equipment or pretreatment, and it can be repeated a number of times until the adsorbent has reached its maximal adsorbing capacity. Adsorption processes are also economic as they can be carried out in mild conditions, reducing the actual costs to that of the adsorbent, which can be selected accordingly [13,14]. The main features of a good adsorbent are high surface area, high adsorption capacity, short adsorption times, and economic and environmentally-friendly production process.

Metal organic frameworks (MOFs) are organic-inorganic hybrid porous materials characterized by a cage-like structure consisting of an array of metal cations held together by organic linkers [15]. Thanks to their large surface area, tunable structural properties and thermal stability, MOFs have been studied for a range of applications, including catalysis [16], gas storage [17,18], enzyme carriers [19,20], sensing [21], and adsorption [22,23,24]. The adsorption capacities of MOFs toward dyes are remarkable [25]. Tian et al. prepared a water-stable cationic Fe-based metal organic framework (CPM-97-Fe) for the adsorption of both anionic and cationic dyes, with adsorption capacities ranging from 157 to 831 mg/g [26]. There are many types of MOFs and, depending on the material, they can range from low to high cost. The lowest cost materials are those whose synthesis is rapid and requires mild conditions as well as environmentally-friendly solvents and reagents. Recently, Sanchez-Sanchez et al. proposed a facile and rapid method to synthesize a Basolite F300-like Fe-BTC MOF under environmentally and economically sustainable conditions (few minutes at room temperature using water as solvent) [27]. This material, was used as a support for enzyme immobilization [28]. To the best of our knowledge, there are only a few studies about dyes’ adsorption using Fe-BTC [29,30,31,32,33]. While adsorption properties of Fe-BTC are significant (e.g., up to 1105 mg/g of methylene blue) [34], the synthetic procedures used require high temperatures or the use of organic solvents.

**Scheme 1:**
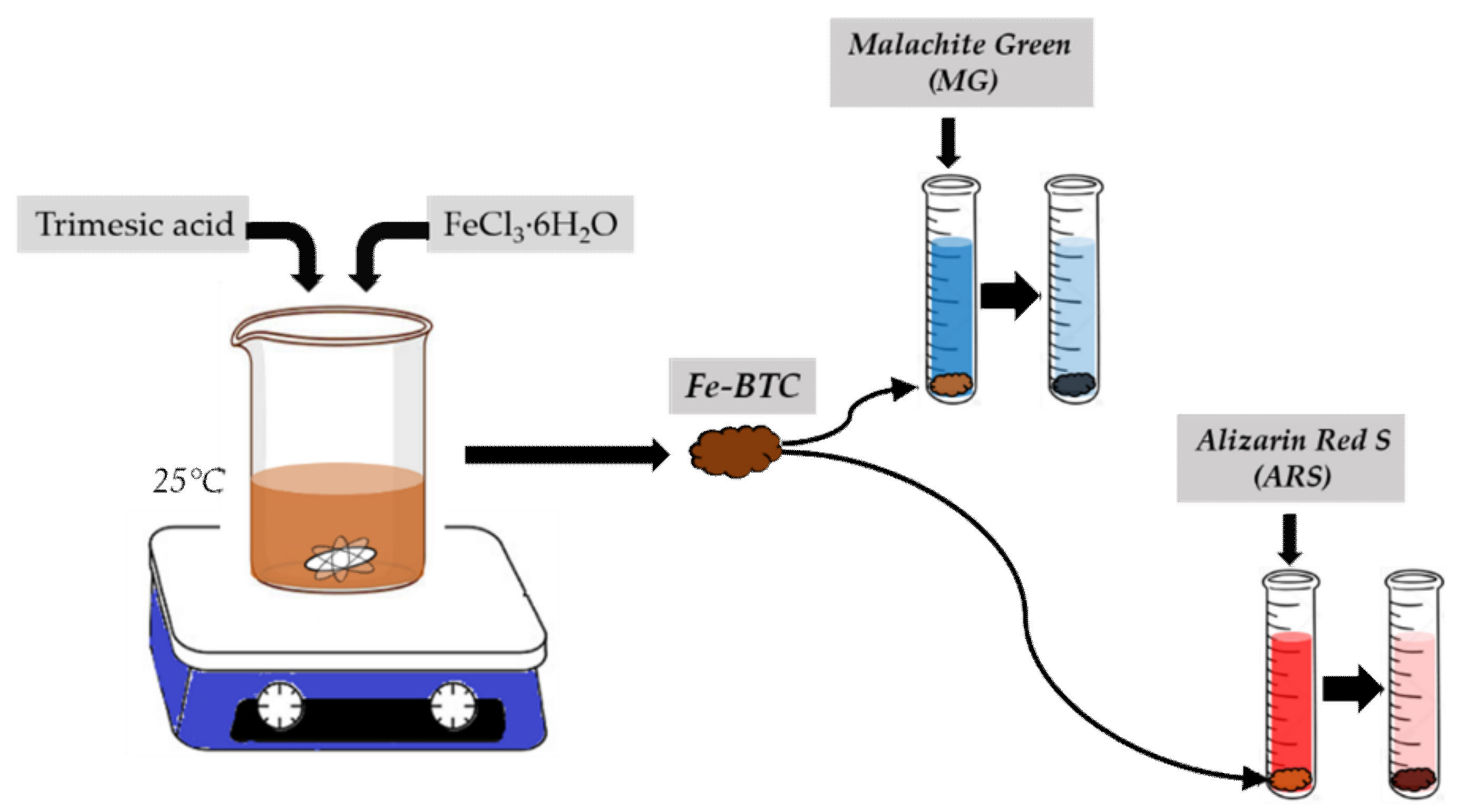
Use of Fe-BTC metal organic framework (MOF) for adsorption of alizarin red (S) and malachite green.

The purpose of this work was to examine the adsorption properties of a Fe-BTC MOF, synthesized according to the method described by Sanchez-Sanchez at al. [27], to remove the anionic dye alizarin red S (ARS) and the cationic dye malachite green (MG) from water (Scheme 1). ARS is a synthetic anthraquinonic acid–base indicator [35,36], used in histology to stain and locate calcium deposits in tissues [37], in geology to identify carbonate minerals, and widely used in textile dyeing. MG is a toxic and carcinogenic triphenylmethane dye, and is widely used in the textile and food industries, as well as in aquaculture as an antifungal, antimicrobial, and antiparasitic agent [38,39,40,41]. The synthesized MOF was characterized by means of XRD (X-ray diffraction), N_2_-adsorption isotherms, SEM (Scanning Electro Microscopy), FTIR (Fourier-Transform Infrared Spectroscopy), TGA (Thermogravimetric Analysis), and ELS (Electrophoretic Light Scattering) techniques. The adsorption kinetics and isotherms of MG and ARS on Fe-BTC MOF were determined in water at room temperature (298 K) by means of UV-Vis spectroscopy to examine the application of Fe-BTC MOF for the removal of toxic dyes from waters.

## 2. Results

### 2.1. Physico-Chemical Characterizations

**Figure 1:**
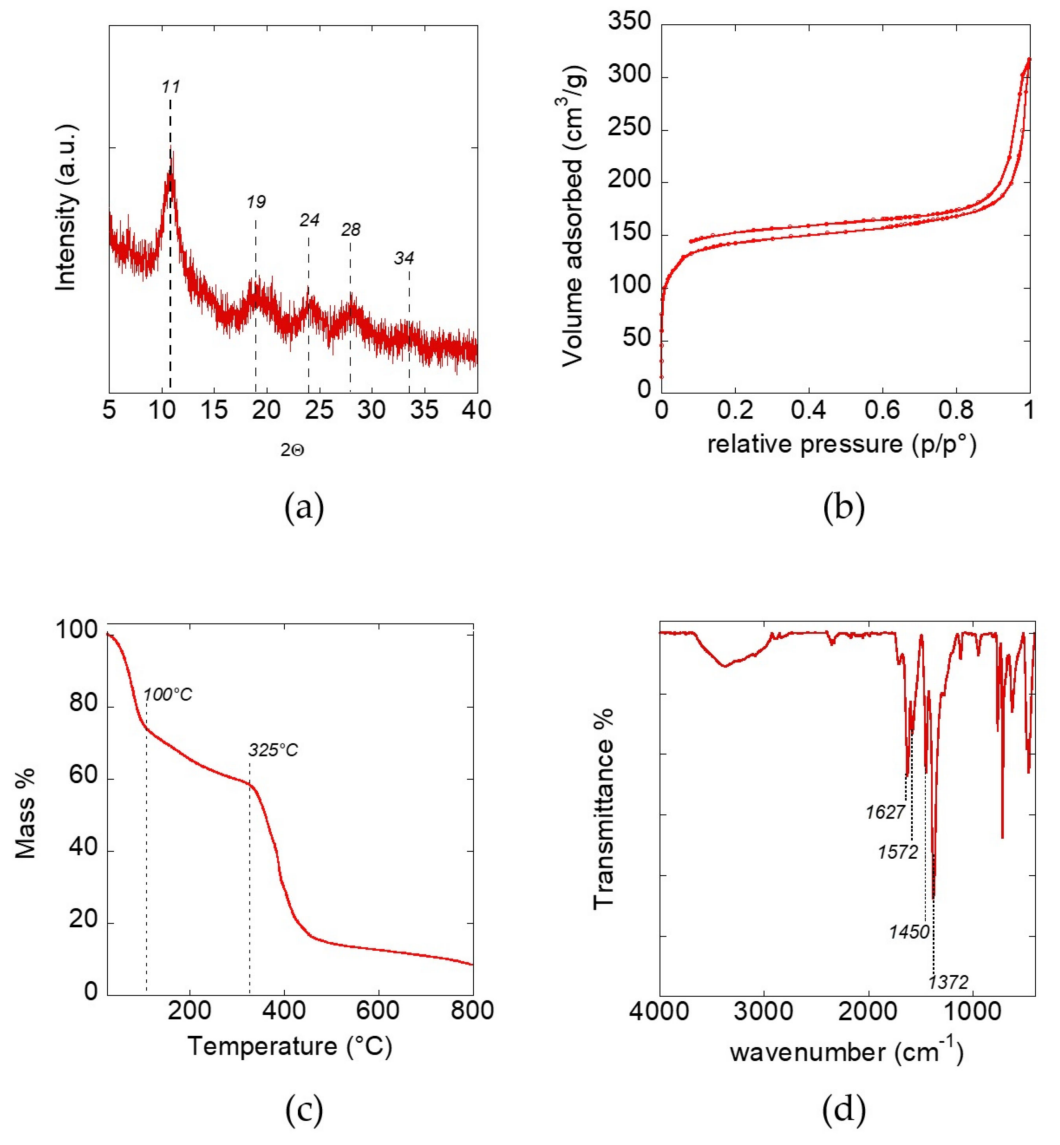
Characterization of Fe-BTC MOF. (**a**) XRD pattern; (**b**) N_2_ physisorption isotherm; (**c**) Thermogravimetric analysis; (**d**) FT-IR spectrum.

Figure 1a shows the XRD pattern of the synthesized Fe-BTC MOF. The pattern is well resolved with peaks at *2Ɵ* = 11°, 19°, 24°, 28° and 34°, in agreement with the literature reports for Fe-BTC MOF [42,43]. The surface area and pore size distribution were obtained by N_2_ adsorption/desorption isotherms (Figure 1b), using the Brunauer–Emmett–Teller (BET) and Barrett–Joyner–Halenda (BJH) methods. The specific surface area was 443 m^2^/*g* and a multi-modal pore size distribution ranged from 4 to 40 nm (Figure S2). Thermogravimetric analysis (Figure 1c) showed a typical two-step decomposition pattern. The initial mass loss at *T* <100 °C is due to the removal of water from the powder. The mass loss in the range 100–325 °C can be attributed to the loss of coordination water [44]. Finally, the mass loss from 325 to 520 °C is ascribed to the decomposition of the organic moiety (trimesic acid) of the MOF [45,46]. The FTIR spectrum of Fe-BTC MOF is shown in Figure 1d. The broad band from 3400 to 3600 cm^−1^ is due to the O-H stretching of adsorbed water. The bands at 1627 and 1572 cm^−1^ and at 1450 and 1372 cm^−1^ are assigned to the asymmetric and the symmetric stretching of the carboxylate groups of Fe-BTC [29,46,47], respectively. The peaks between 770 and 450 cm^−1^ are due to the bending of aromatic C-H bonds.

### 2.2. Effect of pH on Dyes Adsorption on Fe-BTC MOF

**Scheme 2:**
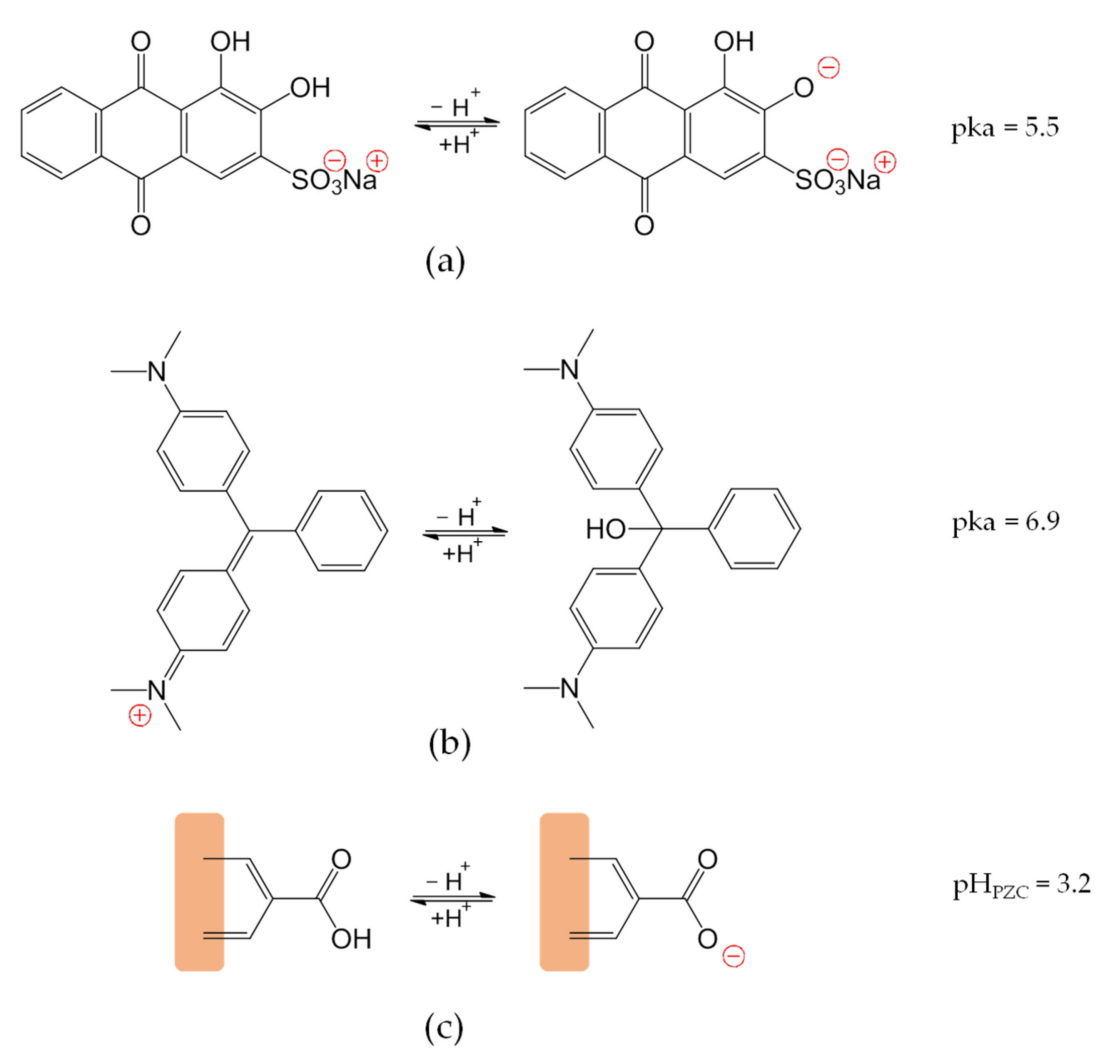
Acid–base equilibria of (**a**) Alizarin red S (ARS) (**b**) Malachite green (MG) and (**c**) Fe-BTC MOF.

The synthesized Fe-BTC MOF was used to adsorb alizarin red S (ARS) and malachite green (MG) from water. Some studies have shown that dye adsorption on MOFs was governed by electrostatic interactions [48]. Thus, it is expected that the pH of the adsorbing solution affects the amount of adsorbed dye as a consequence of the presence/absence of electric charges on both the dye molecules and the adsorbent surface. The pK_a_ of ARS and MG are 5.5 [49] and 6.9 [39], respectively. The former is due to the dissociation of one of the phenolic groups (Scheme 2) [50], and the latter to the conversion of the cation into a carbinol base through addition of OH^−^ (Scheme 2) [51,52].

**Figure 2:**
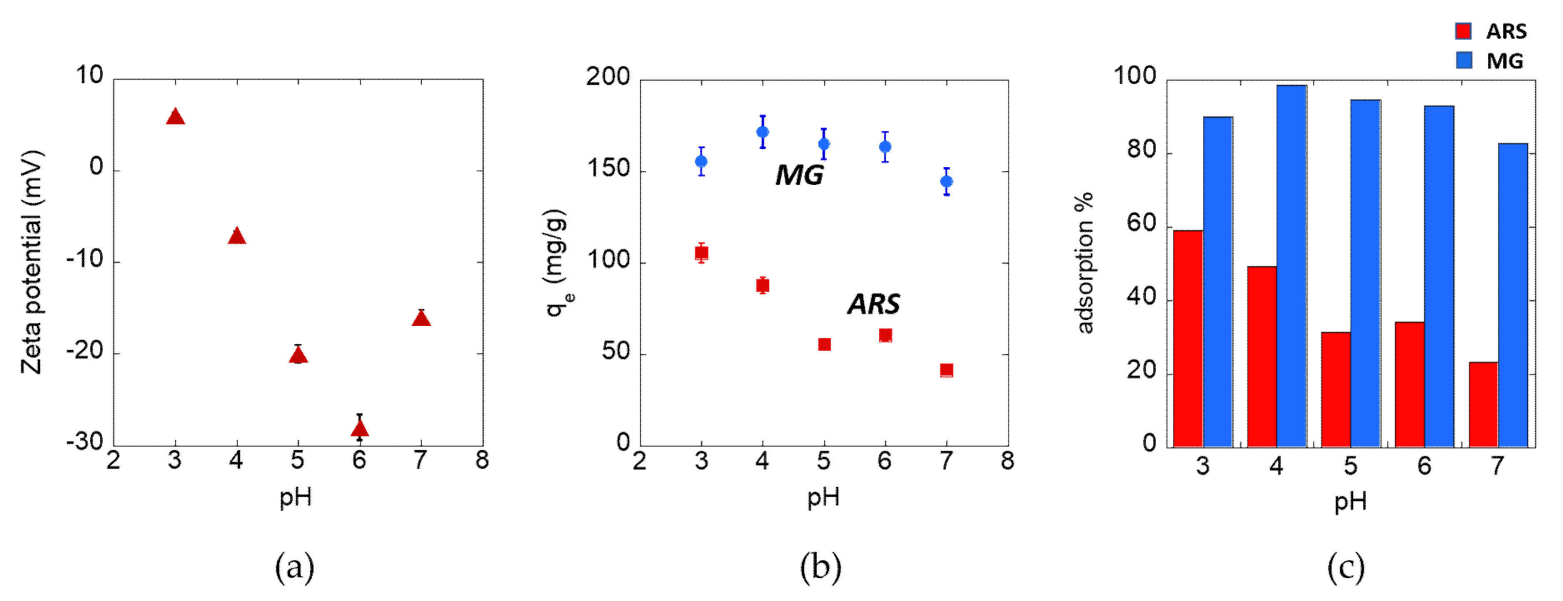
(**a**) Zeta potential of Fe-BTC as a function of pH; (**b**) Adsorption capacity (*q_e_*) of Fe-BTC toward ARS and MG as a function of pH; (**c**) Percentage adsorption of dyes as a function of pH.

The zeta potential of Fe-BTC suspension in water was measured over the pH range 3–7 (Figure 2a and Table S1). Fe-BTC is slightly positive at pH 3 (*ζ* = +8.3 ± 3 mV) and is negatively charged at pH > 4 (*ζ* = −10.3 ± 3 mV) with a *pH_PZC_* (point of zero charge) value of about 3.2 [53], in agreement with the literature [32]. Figure 2b shows the effect of pH on the adsorbed amount at equilibrium (*q_e_*, mg/g) of ARS and MG on Fe-BTC. The *q_e_* values of MG are generally higher than those of ARS. Moreover, while the *q_e_* of MG is unaffected by pH, that of ARS linearly decreases in the pH range 3–7.

Since the *pH_PZC_* (point of zero charge) of Fe-BTC is ca. 3.2 [53], anionic dyes are adsorbed to a lower extent than cationic dyes [54]. Hence, as compared with ARS, higher amounts of MG would be expected to be adsorbed. The two dyes also show different adsorption efficiency (adsorbed amount %) trends (Figure 2c and Table S1) with Fe-BTC possessing a maximum MG adsorption value of 98.5% at pH 4, while the highest value for ARS was 59.1% at pH 3 (Figure 2c). At pH 7, the adsorption capacity was still high for MG (82.9%), but quite low for ARS (23.3%). These trends can be explained by the fact that, at pH 7, MG is neutral, and thus adsorption would predominantly occur via van der Waals forces and would not be affected by electrostatic interactions. Adsorption of ARS on Fe-BTC is not favored at pH 7 as both the dye and the absorbent are negatively charged.

### 2.3. Adsorption Kinetics

**Figure 3:**
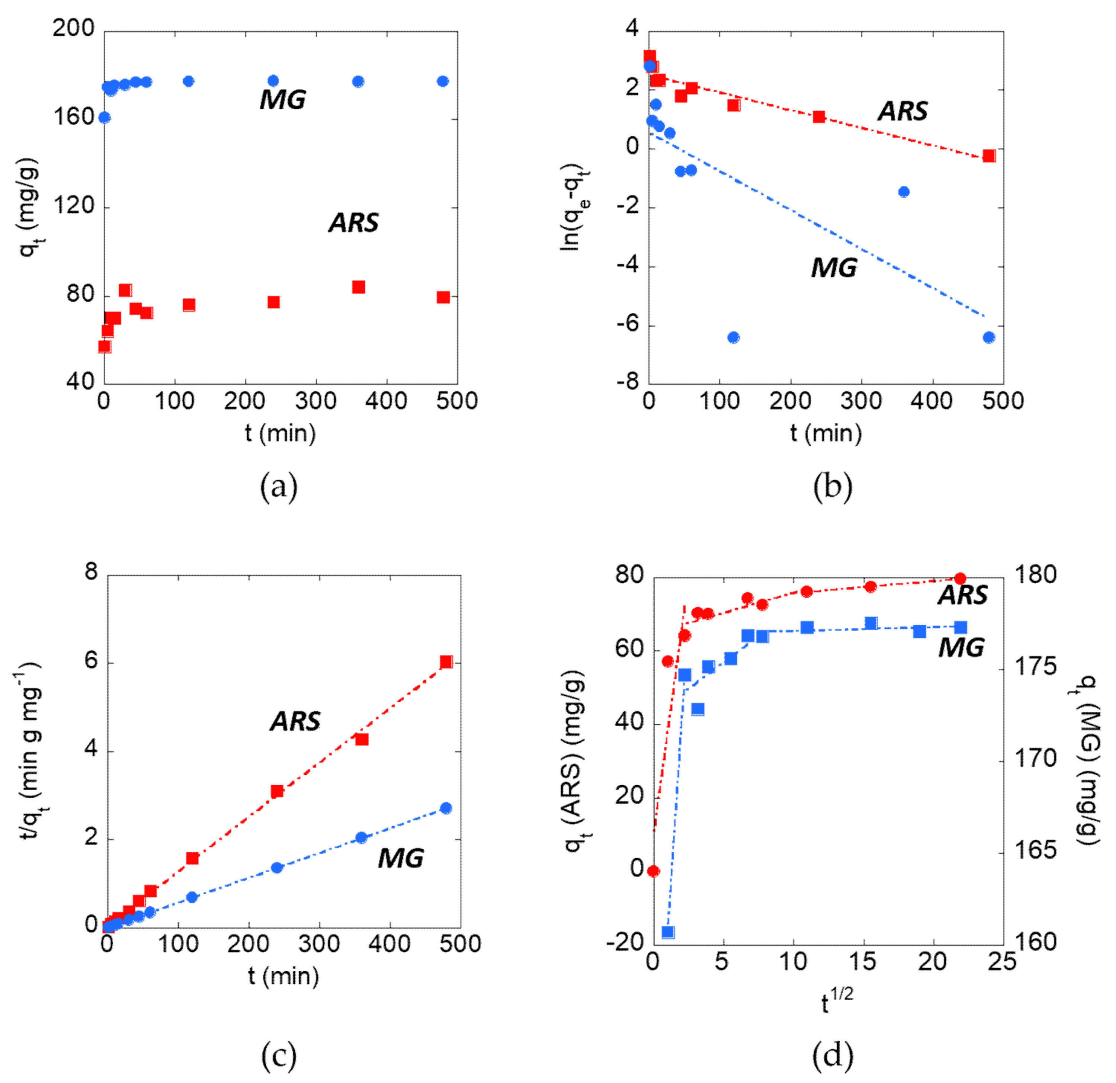
(**a**) Amount of alizarin red S and malachite green adsorbed, *q_t_*, as a function of contact time. The data were fit using linearized kinetics models; (**b**) Pseudo-first order; (**c**) Pseudo-second order; (**d**) Intraparticle diffusion. The experiments were carried out in water at pH = 4 and *T* = 298 K.

The adsorption kinetics of MG and ARS on Fe-BTC MOF were examined in aqueous solution (pH = 4, 298 K). The adsorption process was rapid for both dyes, reaching equilibrium values (corresponding to the plateau in Figure 3a) in 30 min for ARS and 15 min for MG. Under these conditions (*T* = 298 K, pH = 4, initial concentrations of MG and ARS of 1.5 mM), the *q_e_* of MG on Fe-BTC MOF was 177.3 mg/g, while that of ARS reached *q_e_* = 80.4 mg/g. The experimental data were fitted to three different kinetic models, namely, the pseudo-first order (Figure 3b), the pseudo-second order (Figure 3c), and the intraparticle diffusion models (Figure 3d). The kinetic parameters obtained by each model are listed in Table 1. The fitting of the experimental data using the pseudo-first order gave low correlation coefficients (Table 1), thus, demonstrating the inadequacy of this model to describe both ARS and MG adsorption on Fe-BTC. On the contrary, the pseudo-second order model resulted in a very good fitting, as demonstrated by the high correlation coefficients (*R* > 0.99) and a good residuals plot (Figure S3b). Moreover, the values of *q_e_* calculated from pseudo-second order models (177.31 mg/g for MG and 81.09 mg/g for ARS) are very similar to the experimentally observed values (177.28 mg/g for MG and 80.39 mg/g for ARS, Figure 3a). The values of the kinetic constant (*k″*) confirmed that the adsorption process for MG (*k″* = 3.98 × 10^−2^ g∙mg^−1^ min^−1^) was faster than that for ARS (*k″* = 4.29 × 10^−3^ g∙mg^−1^ min^−1^). The fit of the model to the adsorption data demonstrate that the adsorption of the dyes on the adsorbent sites is the rate determining step [29,55]. Figure 3d shows the variation of q_t_ versus t^0.5^ according with the intraparticle diffusion model. The slopes of the three straight lines in Figure 3d represent the kinetic constants of the different steps (1, external diffusion; 2, internal diffusion; and 3, adsorption) involved in the adsorption of ARS and MG dyes on Fe-BTC MOF. However, the fit of this model is of lower quality than that of the pseudo-second order model (Figure S3c), which gives the best description of the obtained kinetic data.

### 2.4. Adsorption Isotherms

**Figure 4:**
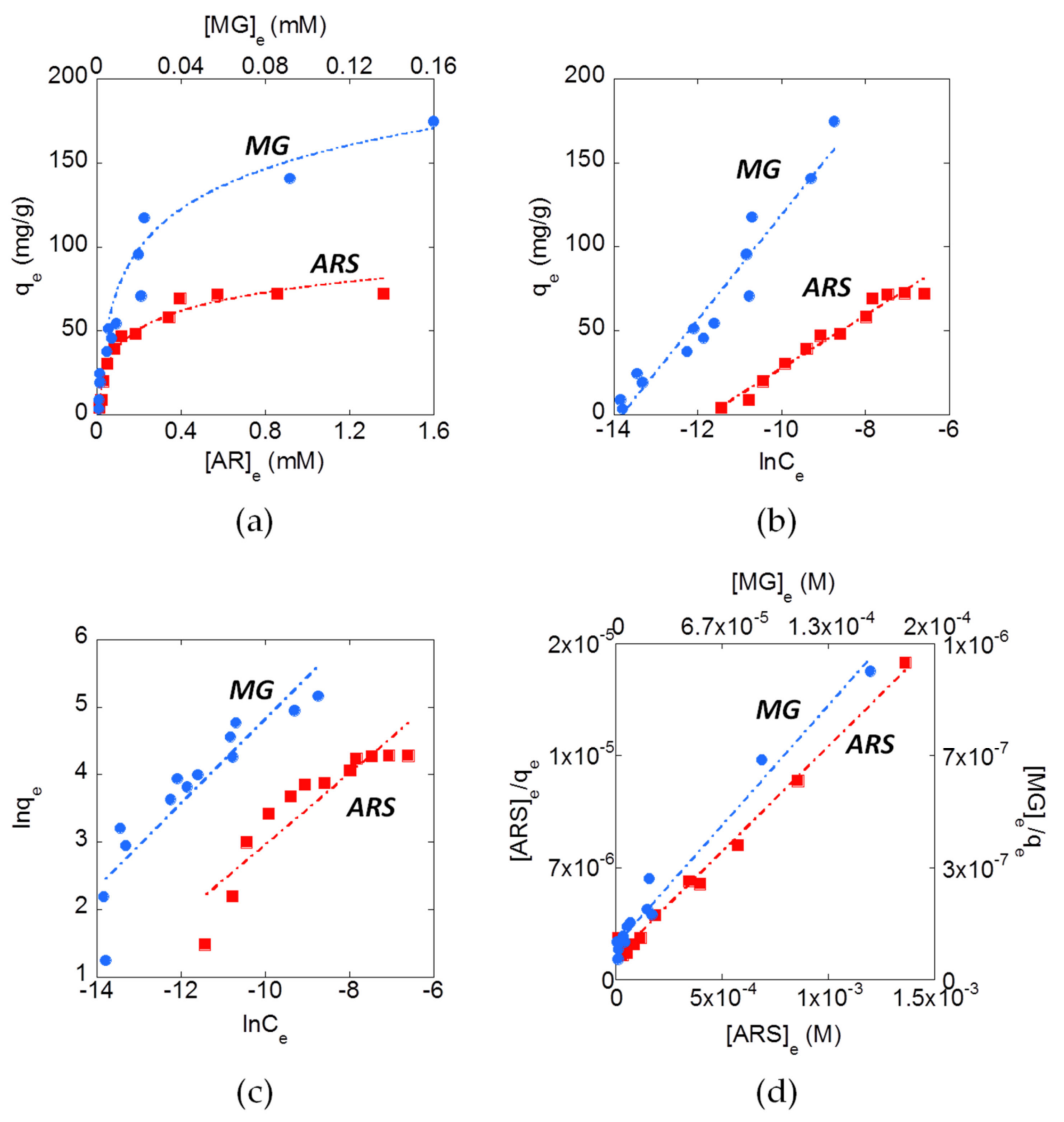
(**a**) Adsorption isotherms of MG and ARS with MOF where *q_e_* is a function of the equilibrium concentration. Adsorption data were fitted using linearized isotherms; (**b**) Temkin; (**c**) Freundlich; (**d**) Langmuir. The experiments were carried out in distilled water for 24 h, at *T* = 298 K.

The adsorption isotherms of ARS and MG on Fe-BTC MOF (*T* = 298 K, pH 4) are shown in Figure 4a. The MOF adsorbed MG to a greater extent than ARS, reaching the maximal adsorbed amounts (*q_e,max_*), corresponding to the isotherm plateaus, *q_e,max_* = 177.3 mg/g and *q_e,max_* = 80.4 mg/g for MG and ARS, respectively. Then, experimental data were tested by applying a fitting procedure based on different linearized isotherm models, namely, Temkin (Figure 4b), Freundlich (Figure 4c), and Langmuir (Figure 4d). The constants associated with each model are reported in Table 2.

By comparing the correlation coefficients (*R)* obtained by applying the different linearized isotherms to the experimental data, with the resulting residual plots (Figure S4), the Langmuir model fits the experimental data better than the other two models. This indicates that a monolayer of adsorbate (dye molecules) was formed on the adsorbent surface (Fe-BTC MOF). Generally, the larger the Langmuir constant *K_L_*, the more favorable the adsorption process [56]. This confirms that adsorption of MG (*K_L_* = 51.56∙10^3^ L/mg) was favored over that of ARS (*K_L_* = 9.30∙10^3^ L/mg) [57]. Then, the Langmuir constant, *K_L_*, was used to calculate the thermodynamic equilibrium constant *K_e_^0^* by means of the equation [58] as:

(1) $$K_{e}^{0}=\frac{K_{L}MM_{Adsorbate}{\left[Adsorbate\right]}^{0}}{\gamma }$$

where *MM_Adsorbate_* is the molecular mass of the adsorbate (*MM_MG_* = 364.91 g mol^−1^ and *MM_ARS_* = 342.26 g mol^−1^), *[Adsorbate]*° is the standard concentration of the adsorbate (1 mol L^−1^), and *γ* is the activity coefficient (dimensionless) that can be considered to have a value of 1 in dilute solution. The *K_e_^0^* values thus calculated were used to determine the standard Gibbs free energy (*ΔG^0^*) for the adsorption process, according to the relationship:

(2) $$\Delta G^{0}=-RTlnK_{e}^{0}$$

where *R* is the universal gas constant (8.314 J K^−1^ mol^−1^) and *T* is the absolute temperature (298.13 K). As shown in Table 2, *ΔG^0^* values were −54.21 kJ mol^−1^ and −58.61 kJ mol^−1^ for the adsorption of ARS and MG, respectively. This indicates that, in standard conditions, the desorption ⇄adsorption equilibrium lies far to the right for both dyes, in agreement with experimental observations.

## 3. Discussion

The adsorption of malachite green on a range of MOFs has been reported [59,60,61,62]. Table 3 summarizes the data from studies relevant to the present work. Among the various types of MOFs tested, the lowest performing in terms of dye adsorption capacity were Cu-BTC [57] and Mil-53-Al-NH_2_ [59]. Both the ZIF-67 prepared by Jin et al. [61] and the UiO-66 prepared by Embaby et al. [55] acted as strong adsorbents with *q_e_* values of 2545 and 400 mg/g, respectively, with adsorption times between 30 and 60 min. The Fe-BTC synthesized by Huo et al. [32] and the mixed-ligand Cu-BDC-BTC compound prepared by Shi et al. [60] had adsorption capacities comparable to those obtained here, 205 and 185 mg/g, respectively, but the time required for the adsorption process (120 min) was four times higher than that obtained by us (30 min).

Li et al. found that the absorption capacity of MIL-53(Al) increased after functionalization with amino groups [59], an increase that can be attributed to hydrogen bond interactions [62] between the amino groups of the dye molecules and the amino groups of MIL-53(Al)-NH_2_; the adsorption capacity achieved by this system is, however, rather low (45.2 mg/g in the case of methylene blue and 37.8 mg/g in the case of malachite green). Jin et al. prepared a ZIF-67 MOF integrated on a polyacrylonitrile membrane to recover the MOF from water solution at the end of the adsorption process [61]. This system had an adsorption capacity of 2545 mg/g of MG, and the time required to complete the process was 60 min. The only study reported to date on the adsorption of alizarin red S by MOFs (Table 3) was carried out by Embaby et al., who reported an adsorption capacity of 400 mg/g for ARS on zirconium-based MOF UiO-66 [55].

Most studies have confirmed that the Langmuir isotherm is the optimal model to describe the adsorption of dyes on the MOF materials described in this study, with the pseudo-second order model representing the best kinetic model. However, in addition to fast kinetics and a high adsorbing capacity, the successful use of an adsorbent for environmental remediation should not be assessed only based on its performance, but also in terms of factors such as cost and ease of preparation. The majority of reports on the use of MOFs utilize synthetic methods that use organic solvents and/or high temperatures. For example, among the adsorbents with higher *q_e_*, the synthesis of UiO-66 was carried out in 1 h in dimethylformamide at 120 °C [55], while Cu-BDC-BTC was prepared in ethanol by heating to 120 °C, for 12 h [60]. The Fe-BTC synthesized by Huo et al., despite being prepared in water, required long synthetic times (12 h) and high temperatures (150 °C in an autoclave) [32]. The most interesting material, both from the point of view of the high adsorbing capacity and of synthesis conditions (25 °C in H_2_O), was the ZIF-67/PAN fibrous membrane proposed by Jin et al. [61]. However, one of the starting reagents of this MOF is the 2-methylimidazole, which is a carcinogenic compound [63,64]. The Fe-BTC used here is significantly easier (and lower cost) to prepare, in an environmentally-friendly manner, with synthesis in less than 1 h at room temperature, using distilled water as the solvent and the reagents, FeCl_3_ and trimesic acid.

## 4. Material and Methods

### 4.1. Chemicals

Tris(hydroxymethyl)-aminomethane (TRIS, ≥99.8%) was purchased from Bio-Rad Laboratories. Iron(III) chloride (97%), sodium hydroxide, trimesic acid, and 4-{[4-(dimethylamino)phenyl](phenyl)methylidene}-*N*,*N*-dimethylcyclohexa-2,5-dien-1-iminium chloride (malachite green) were purchased from Sigma-Aldrich. 3,4-Dihydroxy-9,10-dioxo-2-anthracenesulfonic acid (alizarin red S) was purchased as the sodium salt from Fluka Chemie.

### 4.2. Synthesis and Characterization of Fe-BTC MOF

The Fe-BTC type MOF was prepared following the procedure reported by Sanchez-Sanchez et al. [27,65]. Briefly, 0.3048 g of FeCl_3_ was dissolved in 10.203 mL of distilled water. Then, a solution containing 0.263 g of trimesic acid, 3.685 mL of NaOH 1.06 M, and 6.388 mL of H_2_O was added dropwise under stirring. The solid was collected by filtration under vacuum, washed with distilled water, and dried in air.

X-ray diffraction (XRD) analysis was carried out using an X’PERT Pro PANalytical diffractometer using a Cu K_α_ radiation source. The data were collected from 5 to 40° with a *2θ* step size of 0.013, for 99.19 s. The N_2_ adsorption/desorption isotherms at 77 K were carried out on a ASAP 2020 (Micromeritics) instrument to obtain the surface area (Brunauer–Emmett–Teller, BET) [66] and pore size distribution (Barrett–Joyner–Halenda, BJH) [67]. The FTIR analysis was performed using a Bruker Tensor 27 spectrophotometer equipped with a diamond-ATR accessory and a DTGS detector. A number of 128 scans at a resolution of 2 cm^−1^ were averaged in the spectral range 4000–400 cm^−1^. Thermal analysis data were collected with a STA6000 (Perkin Elmer) thermal analyzer in the 25–850 °C range, under oxygen flow (heating rate = 10 °C/min, flow rate = 40 mL min^−1^). The Zeta potential of Fe-BTC was measured using a Zetasizer Nano ZSP (Malvern Instruments) in backscatter configuration (*θ* = 173°), at a laser wavelength of *λ* = 633 nm, using Zetasizer software (version 7.03) to analyze the data. Zeta potential values were calculated by means of the Henry equation using water as the dispersant medium (*ε_r_* = 78.5 and *η* = 0.89 cP at 25 °C) and *f(κa)* = 1.5 (Smoluchowski approximation). The sample was prepared by suspending Fe-BTC (2 mg/mL) in distilled water adding HCl and NaOH to vary the pH from 3 to 7. Before the measurements the samples were sonicated for 30 min and left stirring overnight. The scattering cell temperature was fixed at 25 °C.

### 4.3. Adsorption Studies

A mass of 100 mg of the synthesized MOF was dispersed in 1 mL of distilled water using a vortex mixer (Figure S1a). To evaluate the optimal pH for the adsorption process, a series of Eppendorf tubes were filled with 1 mL of dye solution and 35 µL of solid dispersion (Figure S1a) at different pH in the range 3–7. The pH was measured using a Metrohm pH-meter and adjusted adding small volumes of HCl and NaOH solutions. All the mixtures were put in a rotating mixer overnight, and then collected by centrifugation (1000 rpm for 1 min). The concentration of dye in the solutions before and after adsorption experiments was determined using a Cary 60 UV-Vis spectrophotometer (Agilent) (*λ* = 516 nm for ARS and 620 nm for MG). The solutions were diluted in Tris-HCl buffer (pH 7, 10 mM) to ensure a constant pH during the measurements, since the absorbance peaks of the dyes, especially in the case of ARS, are influenced by pH [68,69].

Adsorption kinetic studies were carried out analyzing samples withdrawn at different times (from 1 min to 8 h) at a fixed pH of 4 and at a constant concentration of the dyes (1.5 mM). Adsorption isotherms at *T* = 298 K were obtained at constant adsorption time (24 h) and pH (4) at varying initial dye concentrations (from 0.01 to 2 mM).

#### 4.3.1. Adsorption Kinetic Models

The adsorption kinetics were studied by measuring the decrease in concentration of the dyes in solution at given times (*q_t_*) through the following equation,

(3) $$q_{t}=\frac{\left(C_{i}-C_{t}\right)V}{m}$$

where *C_i_* and *C_t_* are the dye concentrations at time = 0 and time = *t*, while *V* and *m* are the volumes of the solution and the mass of the solid, respectively.

The experimental data were fitted using the linearized equations of three different kinetic models. A pseudo-first order model as follows:

(4) $$ln(q_{e}-q_{t})=lnq_{e}-k\prime \cdot t$$

A pseudo-second order model [70,71] as follows:

(5) $$\frac{t}{q_{t}}=\frac{1}{q_{e}^{2}\cdot k\prime \prime }+\frac{t}{q_{e}}$$

and an intraparticle diffusion model [72] as follows:

(6) $$q_{e}=k_{i}\cdot t^{1/2}+x_{i}$$

where *q_e_* is the amount of adsorbed dye at the equilibrium, *k′*, *k″* and *k_i_* are the pseudo-first order constant, pseudo-second order constant, and intraparticle diffusion constant, respectively.

#### 4.3.2. Adsorption Isotherm Models

The adsorption isotherms were obtained by plotting the experimentally adsorbed amounts of dyes, *q_e_*, versus the equilibrium concentration, *C_e_*. The experimental data were fitted through three different isotherm models’, i.e., Temkin (Equation (7)), Freundlich (Equation (8)), and Langmuir (Equation (9)), in their linearized forms [73]:

(7) $$q_{e}=\frac{RT}{b_{T}}lnA_{T}+\frac{RT}{b_{T}}lnC_{e}$$

where *q_e_* is the amount of adsorbed dye at the equilibrium, *q_e,max_* is the maximum monolayer coverage capacity, *b_T_* is the Temkin constant, and *A_T_* is the Temkin equilibrium binding constant.

(8) $$lnq_{e}=lnK_{F}+\frac{1}{n}lnC_{e}$$

where *1/n* (dimensionless) and *K_F_* are the Freundlich constants, the heterogeneity factor, and support capacity (characteristic of each adsorbate-adsorbent pair), respectively.

(9) $$\frac{C_{e}}{q_{e}}=\frac{1}{q_{m}\cdot K_{L}}+\frac{1}{q_{m}}C_{e}$$

where *K_L_* is the Langmuir constant [5,74].

## 5. Conclusions

An Fe-BTC MOF was synthesized following the procedure proposed by Sanchez-Sanchez et al. The structure of the material was characterized by XRD, while its pore diameter distribution (4–40 nm) and surface area (443 m^2^/g) were determined from N_2_ adsorption/desorption isotherms. The zeta potential of aqueous dispersions of Fe-BTC was determined by ELS and a point of zero charge (*pH_pzc_*) of 3.2 was obtained. Further qualitative characterizations were carried out using FTIR and TGA techniques. The data obtained were comparable with those reported in the literature. Then, the Fe-BTC was used as an adsorbent for the removal of two toxic dyes from water, alizarin red S (ARS) and malachite green (MG). The adsorption capacity was measured as a function of time and of the concentration of dye required to obtain the kinetic profiles and the adsorption isotherms of the process, respectively. The adsorption of both dyes was rapid (<30 min) as compared with other reports, which reached equilibrium generally in 60–200 min. The Langmuir model provided the best fit to the adsorption process, with maximum adsorption capacities of 80 and 177 mg/g for ARS and MG on Fe-BTC MOF, respectively. The data obtained for adsorption on to Fe-BTC MOF compare favorably with literature reports. However, what distinguishes this work is the green method used to synthesize the adsorbing material. Indeed, the synthesis of the Fe-BTC MOF was performed in an aqueous solution at room temperature in less than 1 h, unlike the generally used syntheses which require organic solvents or high temperatures and longer times. Furthermore, the adsorption rate of the dyes was higher than most of the other reported MOFs. Future work could be devoted to test the adsorption performance of other toxic dyes or even other classes of toxic substances and to verify the feasibility of continuous processes or on a larger scale. Further work is needed to find the optimal conditions for dye desorption and MOF reuse for multiple adsorption cycles.

## Figures and Tables

**Table 1:** Comparison among different adsorption kinetic models for MG and ARS on Fe-BTC MOF.

	*q_e exp_* (mg g^−1^)	Pseudo-First Order			Pseudo-Second Order			Intraparticle Diffusion		
		*k′* (min^−1^)	*q_e cal_* (mg g^−1^)	*R*	*k″* (g mg^−1^ min^−1^)	*q_e cal_* (mg g^−1^)	*R*	*k_i_* (g mg^−1^ min^−1/2^)	*x_i_* (mg g^−1^)	*R*
ARS	80.39	5.98 × 10^−3^	12.78	0.946	4.29 × 10^−3^	81.09	0.992	27.77 1.10 0.33	10.44 64.81 72.38	0.885 0.873 0.999
MG	177.28	1.3210^−2^	1.75	0.707	3.98 × 10^−2^	177.31	1	11.33 0.58 0.02	149.38 172.51 176.88	1 0.828 0.434

**Table 2:** Comparison among different isotherm models for ARS and MG adsorption on Fe-BTC MOF (pH = 4, *T* = 298 K).

	Temkin			Freundlich			Langmuir			
	*b_T_*	*A_T_* (L mg^−1^)	*R*	*K_F_* (L mg^−1^)	*1/n*	*R*	*K_L_* (L mg^−1^)	*q_e_,_max_* (mg g^−1^)	*R*	*∆G°* (KJ mol^−1^)
ARS	157.59	1.28·10^5^	0.982	3.85·10^3^	0.529	0.910	9.30·10^3^	79.88	0.995	−54.21
MG	79.34	9.87·10^5^	0.967	63.77·10^3^	0.624	0.909	51.56·10^3^	187.24	0.967	−58.61

**Table 3:** Comparison with other systems like that studied in this article.

	Synthesis					Kinetic		Isotherm		
Adsorbent	*T* (°C)	*t* (h)	Solvent	Dye	*q_e(exp)_* (mg/g)	*t* (min)	*k″* (g∙mg min*)*	Model	*K* (L∙mg^−1^)	Ref.
Fe-BTC	25	<1	H_2_O	Alizarin red S	80	30	4.29 × 10^−3^	Langmuir	9.30∙10^3^	This work
				Malachite green	177	30	3.98 × 10^−2^	Langmuir	51.56∙10^3^	
Fe-BTC	150	12	H_2_O	Malachite green	205	120	6.67∙10^−3^	Freundlich	6.49	[32]
UiO-66	120	1	DMF	Alizarin red S	400	36	2.3∙10^−4^	Langmuir	0.06	[55]
Cu-BTC	100	10	EtOH/DMF	Methylene blue	4.68	10	42.39	Langmuir	1.89	[57]
Mil-53(Al) -NH_2_	150	24	DMF/H_2_0	Malachite green	37.8	200	-	Langmuir	0.29	[59]
				Methylene blue	45.2	200	-	Langmuir	0.67	
Cu-BTC/BDC	120	12	EtOH	Malachite green	185	-	-	Freundlich	-	[60]
ZIF-67/PAN	25	<1	H_2_O	Malachite green	2545	60	2.7∙10^−3^	Langmuir	0.05	[61]
NH-ZIF-67	25	<1	MeOH	Malachite green	114.1	240	-	-	-	[62]

## Data Availability

The data presented in this study are available in this paper and supplementary file.

## Supplementary Materials

Supplementary materials can be found at https://www.mdpi.com/1422-0067/22/2/788/s1.

## Abbreviations

MOF	Metal organic frameworks
MG	Malachite green
ARS	Alizarin red S
XRD	X-ray diffraction
FTIR	Fourier transform infrared spectroscopy
TGA	Thermogravimetric analysis
