# Supplementary material for: Adsorption of Malachite Green and Alizarin Red S Dyes Using Fe-BTC Metal Organic Framework as Adsorbent
Source: Int J Mol Sci. 2021 Jan 14;22(2):788. doi: 10.3390/ijms22020788 (PMC7830139; doi:10.3390/ijms22020788)
Supplement: Supplementary file 1 [file ijms-22-00788-s001.pdf]

Supporting information

Adsorption of Malachite Green and Alizarin Red S dyes using Fe-BTC metal organic framework as adsorbent

Giulia Rossella Delpiano[1], Davide Tocco[1], Luca Medda[2], Edmond Magner[3], Andrea Salis[1,4]**

[1] Dipartimento di Scienze Chimiche e Geologiche, Università di Cagliari, Cittadella Universitaria, S.S. 554 bivio Sestu, 09042 Monserrato (CA), Italy; delpiano@unica.it (G.R.D.); davide.tocco@unica.it (D.T.)
[2] Laboratorio NEST Scuola Normale Superiore di Pisa, 56127 Pisa, Italy; medda.luc@gmail.com
[3] Department of Chemical Sciences, Bernal Institute, University of Limerick, Limerick V94 T9PX, Ireland
[4] Consorzio Interuniversitario per lo Sviluppo dei Sistemi a Grande Interfase (CSGI), Unità Operativa University of Cagliari, Monserrato (CA) 09042, Italy

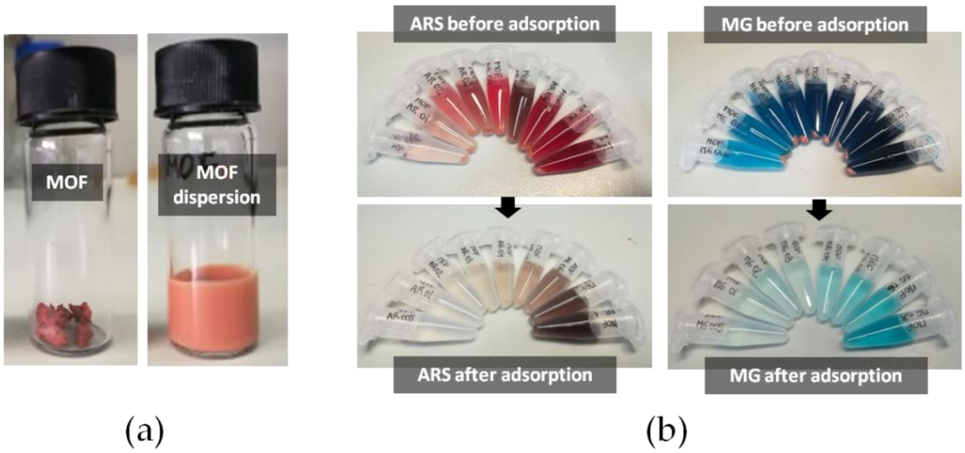

Figure S1 - Images of a) the synthesized MOF before and after dispersion in distilled water; b) Solutions of ARS and MG prior to and after (20 h) adsorption .

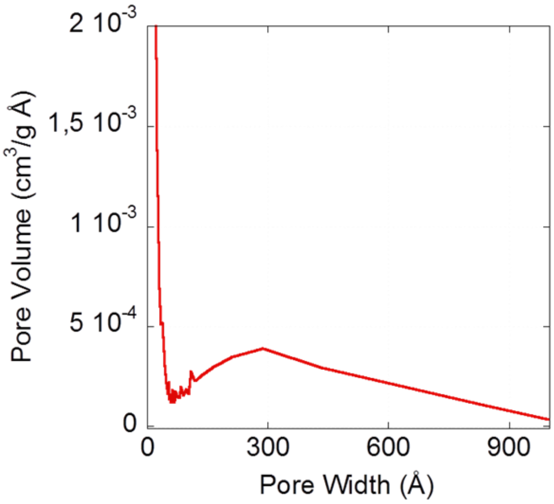

Figure S2 – Pore size distribution (BJH method).

Table S1. Zeta potential of Fe-BTC dispersed in H_2O and the percentage of dyes adsorbed at different pH values (initial concentration of dyes of 1.5 mM).

pH	Fe-BTC Zeta potential (mV)	ARS adsorption%	MG adsorption%
3	+ 6	59.1	89.3
4	- 7	49.2	98.5
5	- 20	31.3	94.7
6	- 28	34.1	93.1
7	-16	23.3	82.9

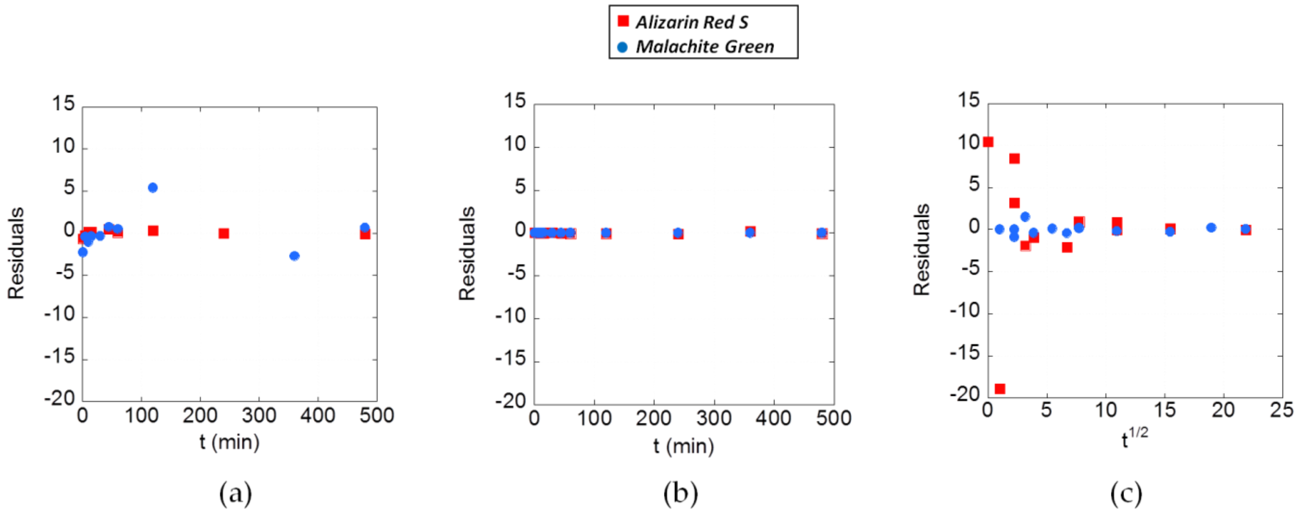

Figure S3 – Residuals graph relative to a) Pseudo-First order, b) Pseudo-Second order and c) Intraparticle diffusion kinetic models.

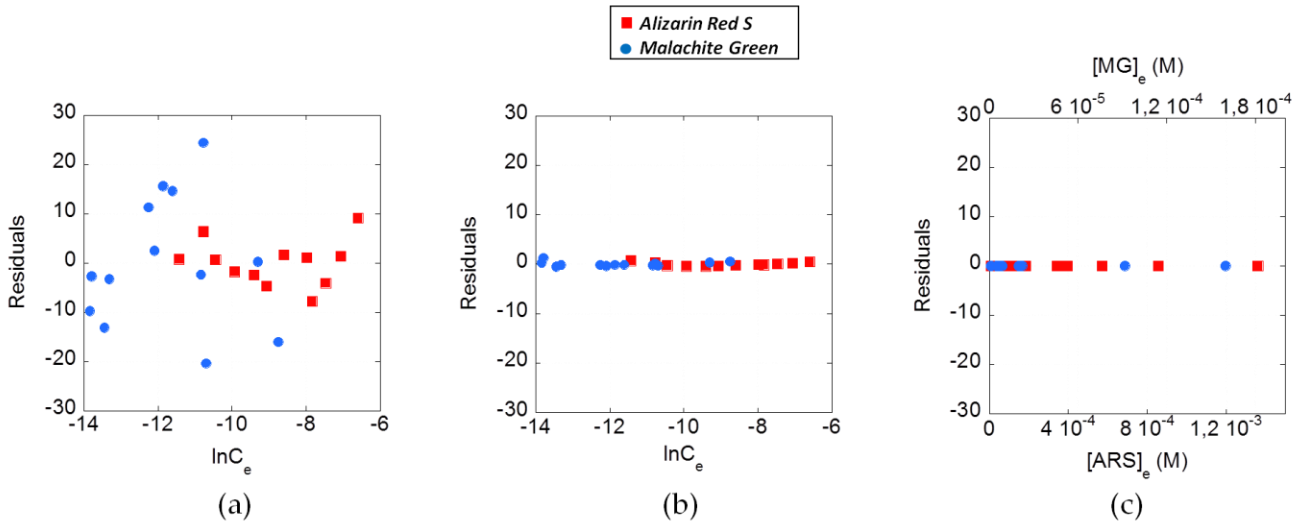

Figure S4 – Residuals graph relative to a) Temkin, b) Freundlich and c) Langmuir isotherms models.
